# New conditions for stability of multiple delayed Cohen-Grossberg Neural Networks of neutral-type

**DOI:** 10.1371/journal.pone.0343312

**Published:** 2026-03-09

**Authors:** Neyir Ozcan

**Affiliations:** Department of Electrical and Electronics Engineering, Faculty of Engineering, Bursa Uludag University, Bursa, Turkey; Brigham Young University, UNITED STATES OF AMERICA

## Abstract

In this research article, we essentially aim to examine the stability properties of a certain type of Cohen-Grossberg neural network. The analysed neural network involves multiple delay parameters. These delay parameters complicate the dynamical behaviour of the system, thereby increasing the risk of oscillations and chaotic behaviour, which adversely affect system stability. However, under specific system parameter constraints, the stability of the system can be ensured. In our study, we developed new adequate stability conditions that guarantee global asymptotic stability for neutral-type Cohen-Grossberg artificial neural networks with multiple delays. These conditions, which can serve as an alternative to the results in the literature, are derived by utilizing suitable Lyapunov functionals and the Lyapunov theorem. The proposed stability conditions are formulated as algebraic equations. Within this context, our proposed stability conditions can be easily examined by using some mathematical methods and software tools. By carrying out a detailed analysis of an instructive numerical example, the results obtained in this article are also shown to establish alternative stability criteria to the corresponding stability conditions given in the past literature.

## 1. Introduction

In recent years, stability analysis of many different types of neural systems has received some particular attention because these neural networks have been proved to possess the abilities to solve different important practical engineering related problems in some particular areas related to some signal and image processing problems, control theory problems, intelligent computational systems, fault diagnosis, pattern recognition, some optimization problems and associative memories applications, (e.g., see [1–5]). When using these neural networks in engineering related applications, one needs to guarantee convergence dynamics of designed neural network models. Because of these reasons, it is crucially important to set up the suitable network parameters which ensure the aimed equilibria properties and stability dynamics of the implemented neural network. In the recent literature, some neural network classes have been adequately applied to real time engineering related problems. In the case of real time applications of neural systems, the convergence dynamics and stability properties may be subject to variation due to the some delay parameters resulting from finite switching rates of electronic circuit components as well as signal transmission times of neurons in neural systems. For instance, introducing delay parameters into the mathematical model of a stable non-delayed neural network may turn this stable neural network into such a neural network that is unstable. Because of such reasons, stability analysis is a critical concept for the dynamical neural systems having some time delay parameters in their mathematically represented models in mathematical neurodynamics of neural systems. Recently, a very large number of the past literature papers have carried out stability investigation of neural networks accepting time delays into state variables of neurons (see, e.g., [6–14]). It should be remarked, at this point that, presenting these time delay parameters to state variables of network neurons may not provide an appropriate modeling approach for dynamical neural networks, as the time derivatives of network neurons state variables can additionally possess some different types of delays. This class of neural networks may show some unexpected, complicated and complex dynamical behaviours. That is why, we must introduce some delays into used mathematical model for these neural systems. Literature defines delays in state variables as time delays, whereas delays in the derivatives of state variables are defined as neutral delays. These type of neural models referred to as neutral-type were successfully employed to be applied to some specific problems including population ecologies, the distributed systems with lossless transmission lines, the propagation-diffusion models [15–17]. Hence, conducting stability of neutral neural networks emerges a crucial importance.

In this research paper, our primary aim is to analyze the convergence dynamics and stability characteristics of Cohen-Grossberg neural systems involved multiple delay parameters. A most commonly used mathematical model for these neural systems is given by the following formulation:


$$\begin{array}{cc} {\dot{x}}_{i}(t)=\; & d_{i}\left(x_{i}(t)\right)(-c_{i}\left(x_{i}(t)\right)+\sum_{j=\text{1}}^{n}a_{ij}f_{j}\left(x_{j}(t)\right)+\sum_{j=\text{1}}^{n}b_{ij}f_{j}\left(x_{j}\left(t-\tau_{ij}\right)\right)+u_{i})\;\\ & \;\;+\sum_{j=\text{1}}^{n}{e_{ij}\dot{x}}_{j}(t-\zeta_{ij}),\;i=\text{1},\;\text{2},\cdots ,\;n \end{array}$$ (1)

In this mathematical model, n represents the number of neurons, the component $x_{i}(t)$ is the state variable of neuron *i*, the components $c_{i}(x_{i}(\text{t}))$ and $d_{i}(x_{i}(\text{t}))$ represent the behaved functions and amplification functions of neurons respectively. $a_{ij}$ and $b_{ij}$ are the neuronal interconnection parameters. These parameters are constant. In the literature, $\tau_{ij}$ and $\zeta_{ij}$ denote time delays and neutral delays respectively and they are also constant. $e_{ij}$ are the fixed coefficients. $f_{i}(x_{i}(t))$ and $u_{i}$ denote nonlinear neuronal activation function for ith neuron and constant external input for ith neuron respectively. In neutral-type neural network represented by equation (1), take $\tau =\text{max}(\tau_{ij})$ and $\zeta =\text{max}\left(\zeta_{ij}\right)$, ∀i, j with μ=max(τ,ζ). Thus, the neural system described as mathematical model (1) possesses initial conditions defined as $x_{i}(t)=\Theta_{i}(t)$ with ${\dot{x}}_{i}(t)=\Delta_{i}(t)$ that will be usually convered in a compact set C([−μ,0], R). It is needed to note that C([−μ,0], R) is a set representing the functions from [−μ,0] to R.

The neural network given in the form described by (1) has $n^{\text{2}}$ time delays and $n^{\text{2}}$ neutral delays. Because of these delay parameters, neural system (1) is said to have multiple delays.

Various subclasses of the neural system (1) can be stated, where the neural system has fewer delay parameters. A neural network that involves n time delays and n neutral delays is said to exhibit discrete delay terms and is modeled by the following mathematical expression:


$${\dot{x}}_{i}(t)=d_{i}\left(x_{i}(t)\right)\left(-c_{i}\left(x_{i}(t)\right)+\sum_{j=\text{1}}^{n}a_{ij}f_{j}\left(x_{j}(t)\right)+\sum_{j=\text{1}}^{n}b_{ij}f_{j}\left(x_{j}\left(t-\tau_{j}\right)\right)+u_{i}\right)+\sum_{j=\text{1}}^{n}{e_{ij}\dot{x}}_{j}\left(t-\zeta_{j}\right),\;i=\text{1},\;\text{2},\cdots ,\;n$$ (2)

The following vector and matrix representation can be used to express the delayed neural system defined by (2):


$$\dot{x}(t)=D\left(x(t)\right)\left(-C\left(x(t)\right)+Af\left(x(t)\right)+Bf\left(x(t-\tau )\right)+u\right)+E\dot{x}(t-\zeta )$$ (3)

in which $\tau_{j}$ are some discrete time delays, $\zeta_{j}$ are the discrete neutral delays, $A=(a_{ij})$ B=($b_{ij})$ and $E=(e_{ij})$ represent the system matrices, $u={\left(u_{\text{1}},\;u_{\text{2}},\cdots ,\;u_{n}\right)}^{T}$, the vector $x(t)={\left(x_{\text{1}}(t),\;x_{\text{2}}(t),\cdots ,\;x_{n}(t)\right)}^{T}$ is the state variables of neural systems (2), the state vector involving time delays is represented by $x(t-\tau )={\left(x_{\text{1}}(t-\tau_{\text{1}}),\;x_{\text{2}}(t-\tau_{\text{2}}),\cdots ,\;x_{n}(t-\tau_{n})\right)}^{T}$, $\dot{x}(t-\zeta )={\left({\dot{x}}_{\text{1}}\left(t-\zeta_{\text{1}}\right),\;{\dot{x}}_{\text{2}}\left(t-\zeta_{\text{2}}\right),\;\cdots ,{\dot{x}}_{n}\left(t-\zeta_{n}\right)\right)}^{T}$, $D(x(t))=diag(d_{i}\left(x_{i}(t)\right)>\text{0})$ and $C(x(t))={\left(c_{\text{1}}(x_{\text{1}}(t)),\;c_{\text{2}}(x_{\text{2}}(t)),\cdots ,c_{n}(\;x_{n}(t))\right)}^{T}$.

If a neural system possesses only one time delay parameter and only one neutral delay term, then this neural network is said to have two delays. Then, a Cohen-Grossberg neural network involving such delay terms will have the mathematical model given by:


$${\dot{x}}_{i}(t)=d_{i}\left(x_{i}(t)\right)\left(-c_{i}\left(x_{i}(t)\right)+\sum_{j=\text{1}}^{n}a_{ij}f_{j}\left(x_{j}(t)\right)+\sum_{j=\text{1}}^{n}b_{ij}f_{j}\left(x_{j}(t-\tau )\right)+u_{i}\right)+\sum_{j=\text{1}}^{n}{e_{ij}\dot{x}}_{j}(t-\zeta ),\;\forall i\;$$ (4)

In formulation (4), the parameter τ represents a fixed time delay, while the term ζ denotes a fixed neutral delay. Neutral neural system defined by the equation (4) can be written as


$$\dot{x}(t)=D\left(x(t)\right)\left(-C\left(x(t)\right)+Af\left(x(t)\right)+Bf\left(x(t-\tau )\right)+u\right)+E\dot{x}(t-\zeta )$$ (5)

with the new time delayed system vector of having a form $x(t-\tau )={\left(x_{\text{1}}(t-\tau ),\;x_{\text{2}}(t-\tau ),\cdots ,\;x_{n}(t-\tau )\right)}^{T}$ and the new neutral delayed system state vector of having a form $\dot{x}(t-\zeta )={\left({\dot{x}}_{\text{1}}(t-\zeta ),\;{\dot{x}}_{\text{2}}(t-\zeta ),\;\cdots ,{\dot{x}}_{n}(t-\zeta )\right)}^{T}$.

Network functions denoted by $d_{i}(x_{i}(t))$ and $c_{i}(x_{i}(t))$ and activation functions denoted by $f_{i}(x_{i}(t))$ have the nonlinear form. Some basic properties of these nonlinear functions are generally stated by the following conditions:

$H_{\text{1}}$: The amplification functions $d_{i}(x)$ hold the following conditions


$$\text{0}<v_{i}\leq d_{i}(x)\leq \phi_{i},\;\forall x\in R,\;\forall i$$

with $v_{i}$ and $\phi_{i}$ are being some real positive constants.

$H_{\text{2}}$: The behaved functions $c_{i}(x)$ hold the following conditions

$\text{0}<\gamma_{i}(x-y{)}^{\text{2}}\leq \left|c_{i}(x)-c_{i}(y)\right||x-y|\leq \psi_{i}(x-y{)}^{\text{2}},\;\forall x,\;y\in R,\;x\neq y,\;\forall i$.

with $\gamma_{i}$ and $\psi_{i}$ are being some real positive constants.

$H_{\text{3}}$: The activation functions $f_{i}(x)$ hold the following conditions


$$\left|f_{i}(x)-f_{i}(y)\leq {\text{𝓁}}_{i}\right||x-y|,\;\forall x,\;y\in R,\;\forall i$$

with ${\text{𝓁}}_{i}$ are being some real positive constants.

Since neural networks models (2) and (4) can be directly put in some suitable vector-matrix forms, it is relatively easier to study stability analysis of these systems when compared with stability analysis of neural system (1). In the past literature, various techniques, methods and approaches have been employed to determine various useful and sufficient criteria for stability of neural network models given by (2) and (4). In [18] and [19], the integral inequality techniques, in [20], general delay partitioning techniques and the Jensen inequality, in [21], the semimartingale convergence technique, in [22], the delay partitioning method, in [23], functionals of triple or quadruple integral terms, in [24] the Wirtinger –type integral inequalities and some convex combination techniques, in [25], Leibniz-Newton formula principle, in [26], the stochastic analysis theory principle, In [27], the descriptor transformation theory method has been combined with Lyapunov stability theorems. In addition to these stability conditions which are defined by different forms of linear matrix inequalities (LMI), some stability conditions of neural network models (2) and (4) in algebraic forms have also been presented in [28–37].

The first results regarding global stability of neutral neural network models having multiple delay terms have been given in [38]. In this study, neutral-type Hopfield neural networks have been analyzed. We also note here that when the amplification functions $d_{i}(x_{i}(t))$ are equal to 1 for all *i*, and for some positive constants $c_{i}$, $c_{i}(x_{i}(t))=c_{i}x_{i}(t)$ for all *i*, the multiple delayed neutral neural system stated in (1) will take the form of a delayed-type Hopfield neural network. Hence, the author of [38] has initiated the area of stability issues for neutral neural network models admitting multiple delay parameters. Then, motivated by the results of [38], some other researchers have made some important contributions to the investigation of stability conditions of systems (1). In [39–43], various sets of criteria for stability of neural system (1) have been presented. In this new research article, we will conduct and alternative Lyapunov stability analysis of neural network model (1) and obtain new alternative conditions that ensure stability of this neural systems.

## 2. Stability analysis

We will proceed, in this section, with obtaining new results which assure global asymptotic stability of neural network whose mathematical representation is stated in (1). For this objective, one can find it to be helpful to transfer equilibrium points that are possessed by neutral neural system given in (1) to the origin. If the constant vector denoted by $\hat{x}$ whose components are ${\hat{x}}_{\text{1}},\;{\hat{x}}_{\text{2}},\;\cdots ,\;{\hat{x}}_{n}$, represents a fixed equilibrium point of the neural network whose dynamics is described in (1), then the simple equation $z_{i}(t)=x_{i}(t)-{\hat{x}}_{i}$ will directly transform the equilibrium point of the system (1) to the origin. By assigning $z_{i}(t)$ to the difference $x_{i}(t)-{\hat{x}}_{i},\;\forall i$, in equation (1), a new neutral-type neural system is obtained, and dynamical behaviour of this system can be defined using the following mathematical equation:


$${\dot{z}}_{i}(t)=\propto_{i}\left(z_{i}(t)\right)\left(-\beta_{i}\left(z_{i}(t)\right)+\sum_{j=\text{1}}^{n}a_{ij}g_{j}\left(z_{j}(t)\right)+\sum_{j=\text{1}}^{n}b_{ij}g_{j}\left(z_{j}\left(t-\tau_{ij}\right)\right)\;\right)+\sum_{j=\text{1}}^{n}{e_{ij}\dot{z}}_{j}(t-\zeta_{ij}),\;\forall i$$ (6)

In the system given by (6), the transformed nonlinear strictly positive system functions $\propto_{i}(z_{i}(t))$ and the transformed nonlinear strictly increasing system functions $\beta_{i}(z_{i}(t))$ are obtained to be in the forms $\propto_{i}(z_{i}(\text{t}))=d_{i}(z_{i}(t)+{\hat{x}}_{i})$ and $\beta_{i}(z_{i}(t))=c_{i}\left(z_{i}(t)+{\hat{x}}_{i}\right)-c_{i}({\hat{x}}_{i})$ respectively. Finally, it is stated that the transformed nonlinear activation functions $g_{i}(z_{i}(t))$ are expressed as $g_{i}(z_{i}(t))=f_{i}\left(z_{i}(t)+{\hat{x}}_{i}\right)-f_{i}({\hat{x}}_{i})$. Considering original constraints conditions on system functions, it is obvious that the following conditions will be satisfied by these new functions under conditions $H_{\text{1}},\;H_{\text{2}}$ and $H_{\text{3}}$:


$${\tilde{H}}_{\text{1}}:\text{0}<v_{i}\leq \propto_{i}\left(z_{i}(t)\right)\leq \phi_{i},\;\forall i$$

$$\begin{array}{c} {\tilde{H}}_{\text{2}}:\gamma_{i}z_{i}^{\text{2}}(t)\leq \beta_{i}\left(z_{i}(t)\right)z_{i}(t)\leq \psi_{i}z_{i}^{\text{2}}(t),\;\forall i \end{array}$$

$${\tilde{H}}_{\text{3}}:\left|g_{i}\left(z_{i}(t)\right)\right|\leq {\text{𝓁}}_{i}\left|z_{i}(t)\right|,\;\forall i$$

In the proceeding theorem, we derive the major results of our paper:

### 2.1. Theorem 1

For the system (6), assume that the system functions justify assumptions ${\tilde{H}}_{\text{1}},\;{\tilde{H}}_{\text{2}}$ and ${\tilde{H}}_{\text{3}}$. In this case, the transformed neural system expressed by (6) is globally asymptotically stable if there exist real positive numbers $\varepsilon_{\text{1}}>\text{0},\;\varepsilon_{\text{2}}>\text{0},\;\varepsilon_{\text{3}}>\text{0}$ and $\varepsilon_{i}>\text{0},\;\forall i$ such that the criteria given below are satisfied.


$$\delta_{i}=\text{2}\xi \in_{i}\frac{\gamma_{i}}{{\text{𝓁}}_{i}}-\sum_{j=\text{1}}^{n}(\frac{\xi }{\varepsilon_{\text{1}}}\in_{i}\left|a_{ij}\right|+\varepsilon_{\text{1}}\in_{j}\left|a_{ji}\right|)-\sum_{j=\text{1}}^{n}(\frac{\xi }{\varepsilon_{\text{2}}}\in_{i}\left|b_{ij}\right|+\varepsilon_{\text{2}}\in_{j}\left|b_{ji}\right|)-\frac{\xi }{\varepsilon_{\text{3}}}\sum_{j=\text{1}}^{n}\frac{\in_{i}}{v_{i}}\left|e_{ij}\right|>\text{0},\;\forall i$$ (7)

and


$$\begin{array}{c} \kappa_{i}=\text{2}(\text{1}-\xi )\frac{\in_{i}}{\phi_{i}}-\frac{\text{1}-\xi }{\varepsilon_{\text{1}}}\in_{i}\sum_{j=\text{1}}^{n}\left|a_{ij}\right|-\frac{\text{1}-\xi }{\varepsilon_{\text{2}}}\in_{i}\sum_{j=\text{1}}^{n}\left|b_{ij}\right|-\frac{\text{1}-\xi }{\varepsilon_{\text{3}}}\sum_{j=\text{1}}^{n}\frac{\in_{i}}{v_{i}}\left|e_{ij}\right|-\varepsilon_{\text{3}}\sum_{j=\text{1}}^{n}\frac{\in_{j}}{v_{j}}\left|e_{ji}\right|>\text{0},\;\forall i \end{array}$$ (8)

where 0<ξ<1.

**Proof:**

Construct three different functionals:


$$\;V_{\text{1}}(t)=\text{2}\sum_{i=\text{1}}^{n}\int_{\text{0}}^{z_{i}(t)}\in_{i}{\text{𝓁}}_{i}\frac{s}{\propto_{i}(s)}ds$$ (9)

$$V_{\text{2}}(t)=\text{2}\sum_{i=\text{1}}^{n}\in_{i}\int_{\text{0}}^{z_{i}(t)}\beta_{i}(s)ds+\text{2}\sum_{i=\text{1}}^{n}\in_{i}\int_{\text{0}}^{t}\frac{{\dot{z}}_{i}^{\text{2}}(\theta )}{\propto_{i}(z_{i}(\theta ))}d\theta -\text{2}\sum_{i=\text{1}}^{n}\in_{i}\int_{\text{0}}^{t}\frac{{\dot{z}}_{i}^{\text{2}}(\theta )}{\propto_{i}(z_{i}(\theta ))}d\theta$$ (10)

$$V_{\text{3}}(t)=\sum_{i=\text{1}}^{n}\sum_{j=\text{1}}^{n}\left(\varepsilon_{\text{2}}\int_{t-\tau_{ji}}^{t}\epsilon_{j}\left|b_{ji}\right|{\text{𝓁}}_{i}^{\text{2}}z_{i}^{\text{2}}(\xi )d\xi +\varepsilon_{\text{3}}\int_{t-\zeta_{ji}}^{t}\frac{\epsilon_{j}}{v_{j}}e_{ji}{\dot{z}}_{i}^{\text{2}}(\varphi )d\varphi \right)\;+\sum_{i=\text{1}}^{n}\sum_{j=\text{1}}^{n}\left(\frac{p}{n}\int_{t-\tau_{ji}}^{t}z_{i}^{\text{2}}(\xi )d\xi +\frac{q}{n}\int_{t-\zeta_{ji}}^{t}{\dot{z}}_{i}^{\text{2}}(\varphi )d\varphi \right)$$ (11)

In the equation (11), *p* and *q* are positive constants. These values will be determined as needed.

Now, construct a positive real valued Lyapunov functional for neural system expressed by equation (6):


$$V(t)=\xi V_{\text{1}}(t)+(\text{1}-\xi )V_{\text{2}}(t)+V_{\text{3}}(t)$$ (12)

For $V_{\text{1}}(t)$, which is one of the functions within the constructed Lyapunov functional, ${\dot{V}}_{\text{1}}(t)$ is calculated and the following mathematical equation is obtained:


$$\;{\dot{V}}_{\text{1}}(t)=\text{2}\sum_{i=\text{1}}^{n}\in_{i}{\text{𝓁}}_{i}z_{i}(t)\frac{{\dot{z}}_{i}(t)}{\alpha_{i}\left(z_{i}(t)\right)}$$ (13)

$$=\text{2}\sum_{i=\text{1}}^{n}\in_{i}{\text{𝓁}}_{i}z_{i}(t)\left(-\beta_{i}\left(z_{i}(t)\right)+\sum_{j=\text{1}}^{n}a_{ij}g_{j}\left(z_{j}(t)\right)+\sum_{j=\text{1}}^{n}b_{ij}g_{j}\left(z_{j}\left(t-\tau_{ij}\right)\right)\right)$$

$$\;+\text{2}\sum_{i=\text{1}}^{n}\sum_{j=\text{1}}^{n}\frac{\in_{i}{\text{𝓁}}_{i}}{\alpha_{i}(z_{i}(t))}e_{ij}z_{i}(t){\dot{z}}_{j}\left(t-\zeta_{ij}\right)$$

$$\;=-\text{2}\sum_{i=\text{1}}^{n}\in_{i}{\text{𝓁}}_{i}\beta_{i}\left(z_{i}(t)\right)z_{i}(t)+\text{2}\sum_{i=\text{1}}^{n}\sum_{j=\text{1}}^{n}\in_{i}{\text{𝓁}}_{i}a_{ij}g_{j}\left(z_{j}(t)\right)z_{i}(t)$$

$$\;+\text{2}\sum_{i=\text{1}}^{n}\sum_{j=\text{1}}^{n}\in_{i}{\text{𝓁}}_{i}b_{ij}g_{j}\left(z_{j}\left(t-\tau_{ij}\right)\right)z_{i}(t)+\text{2}\sum_{i=\text{1}}^{n}\sum_{j=\text{1}}^{n}\frac{\in_{i}{\text{𝓁}}_{i}}{\alpha_{i}(z_{i}(t))}e_{ij}z_{i}(t){\dot{z}}_{j}\left(t-\zeta_{ij}\right)$$

Condition ${\tilde{H}}_{\text{2}}$ ensures that $\beta_{i}\left(z_{i}(t)\right)z_{i}(t)\geq \gamma_{i}z_{i}^{\text{2}}(t)$. Thus, (13) leads to


$$\;{\dot{V}}_{\text{1}}(t)\leq -\text{2}\sum_{i=\text{1}}^{n}\in_{i}{\text{𝓁}}_{i}\gamma_{i}z_{i}^{\text{2}}(t)+\text{2}\sum_{i=\text{1}}^{n}\sum_{j=\text{1}}^{n}\in_{i}{\text{𝓁}}_{i}a_{ij}g_{j}\left(z_{j}(t)\right)z_{i}(t)$$

$$\;+\text{2}\sum_{i=\text{1}}^{n}\sum_{j=\text{1}}^{n}\in_{i}{\text{𝓁}}_{i}b_{ij}g_{j}\left(z_{j}\left(t-\tau_{ij}\right)\right)z_{i}(t)+\text{2}\sum_{i=\text{1}}^{n}\sum_{j=\text{1}}^{n}\frac{\in_{i}{\text{𝓁}}_{i}}{\alpha_{i}(z_{i}(t))}e_{ij}z_{i}(t){\dot{z}}_{j}\left(t-\zeta_{ij}\right)$$ (14)

For $V_{\text{2}}(t)$, which is another function within the constructed Lyapunov functional, ${\dot{V}}_{\text{2}}(t)$ is calculated:


$${\dot{V}}_{\text{2}}(t)=\text{2}\sum_{i=\text{1}}^{n}\left(\in_{i}\beta_{i}\left(z_{i}(t)\right){\dot{z}}_{i}(t)+\in_{i}\frac{{\dot{z}}_{i}^{\text{2}}(t)}{\alpha_{i}(z_{i}(t))}-\in_{i}\frac{{\dot{z}}_{i}^{\text{2}}(t)}{\alpha_{i}(z_{i}(t))}\right)$$ (15)

Note that


$$\text{2}\sum_{i=\text{1}}^{n}\in_{i}\frac{{\dot{z}}_{i}^{\text{2}}(t)}{\alpha_{i}\left(z_{i}(t)\right)}=\text{2}\sum_{i=\text{1}}^{n}\left(-\in_{i}\beta_{i}\left(z_{i}(t)\right)+\sum_{j=\text{1}}^{n}\left(\in ia_{ij}g_{j}\left(z_{j}(t)\right)+\in ib_{ij}g_{j}\left(z_{j}\left(t-\tau_{ij}\right)\right)\right){\dot{z}}_{i}(t)\right)$$ (16)

$$\;+\text{2}\sum_{i=\text{1}}^{n}\sum_{j=\text{1}}^{n}\frac{\in_{i}}{\alpha_{i}(z_{i}(t))}e_{ij}{\dot{z}}_{i}(t){\dot{z}}_{j}\left(t-\zeta_{ij}\right)$$

Inserting [16] into [15] results in:


$${\dot{V}}_{\text{2}}(t)=\text{2}\sum_{i=\text{1}}^{n}\sum_{j=\text{1}}^{n}\left(\;\in ia_{ij}{\dot{z}}_{i}(t)g_{j}\left(z_{j}(t)\right)+\in ib_{ij}{\dot{z}}_{i}(t)g_{j}\left(z_{j}\left(t-\tau_{ij}\right)\right)+\frac{\in i}{\alpha_{i}(z_{i}(t))}e_{ij}{\dot{z}}_{i}(t){\dot{z}}_{j}\left(t-\zeta_{ij}\right)\right)-\text{2}\sum_{i=\text{1}}^{n}\in_{i}\frac{{\dot{z}}_{i}^{\text{2}}(t)}{\alpha_{i}(z_{i}(t))}$$ (17)

$$\;\leq \text{2}\sum_{i=\text{1}}^{n}\sum_{j=\text{1}}^{n}\left(\in ia_{ij}{\dot{z}}_{i}(t)g_{j}\left(z_{j}(t)\right)+\in_{i}b_{ij}{\dot{z}}_{i}(t)g_{j}\left(z_{j}\left(t-\tau_{ij}\right)\right)+\frac{\in_{i}}{\alpha_{i}(z_{i}(t))}e_{ij}{\dot{z}}_{i}(t){\dot{z}}_{j}\left(t-\zeta_{ij}\right)\right)-\text{2}\sum_{i=\text{1}}^{n}\frac{\in_{i}}{\phi_{i}}{\dot{z}}_{i}^{\text{2}}(t)$$

One may state the following inequalities that are important in the proofs:


$$\text{2}\sum_{i=\text{1}}^{n}\sum_{j=\text{1}}^{n}\in_{i}{\text{𝓁}}_{i}z_{i}(t)a_{ij}g_{j}\left(z_{j}(t)\right)\leq \text{2}\sum_{i=\text{1}}^{n}\sum_{j=\text{1}}^{n}\in_{i}{\text{𝓁}}_{i}\left|z_{i}(t)\right|\left|a_{ij}\right|\left|g_{j}\left(z_{j}(t)\right)\right|\;$$

$$\;\leq \sum_{i=\text{1}}^{n}\sum_{j=\text{1}}^{n}\left(\frac{\text{1}}{\varepsilon_{\text{1}}}\in_{i}\left|a_{ij}\right|{\text{𝓁}}_{i}^{\text{2}}z_{i}^{\text{2}}(t)+\varepsilon_{\text{1}}\in_{i}\left|a_{ij}\right|g_{j}^{\text{2}}\left(z_{j}(t)\right)\right)\;$$

$$\;\leq \sum_{i=\text{1}}^{n}\sum_{j=\text{1}}^{n}\left(\frac{\text{1}}{\varepsilon_{\text{1}}}\in_{i}\left|a_{ij}\right|{\text{𝓁}}_{i}^{\text{2}}z_{i}^{\text{2}}(t)+\varepsilon_{\text{1}}\in_{i}\left|a_{ij}\right|{\text{𝓁}}_{j}^{\text{2}}z_{j}^{\text{2}}(t)\right)\;$$

$$\;=\sum_{i=\text{1}}^{n}\sum_{j=\text{1}}^{n}\left(\frac{\text{1}}{\varepsilon_{\text{1}}}\in_{i}\left|a_{ij}\right|{\text{𝓁}}_{i}^{\text{2}}z_{i}^{\text{2}}(t)+\varepsilon_{\text{1}}\in_{j}\left|a_{ji}\right|{\text{𝓁}}_{i}^{\text{2}}z_{i}^{\text{2}}(t)\right)$$ (18)

$$\text{2}\sum_{i=\text{1}}^{n}\sum_{j=\text{1}}^{n}\in_{i}b_{ij}{\text{𝓁}}_{i}z_{i}(t)g_{j}\left(z_{j}\left(t-\tau_{ij}\right)\right)\leq \text{2}\sum_{i=\text{1}}^{n}\sum_{j=\text{1}}^{n}\in_{i}{\text{𝓁}}_{i}\left|b_{ij}\right|\left|z_{i}(t)\right|\left|g_{j}\left(z_{j}\left(t-\tau_{ij}\right)\right)\right|$$ (19)

$$\;\leq \sum_{i=\text{1}}^{n}\sum_{j=\text{1}}^{n}\left(\frac{\text{1}}{\varepsilon_{\text{2}}}\in_{i}\left|b_{ij}\right|{\text{𝓁}}_{i}^{\text{2}}z_{i}^{\text{2}}(t)+\varepsilon_{\text{2}}\;\;\in_{i}\left|b_{ij}\right|g_{j}^{\text{2}}\left(z_{j}\left(t-\tau_{ij}\right)\right)\right)$$

$$\;\leq \sum_{i=\text{1}}^{n}\sum_{j=\text{1}}^{n}\left(\frac{\text{1}}{\varepsilon_{\text{2}}}\in_{i}\left|b_{ij}\right|{\text{𝓁}}_{i}^{\text{2}}z_{i}^{\text{2}}(t)+\varepsilon_{\text{2}}\;\in_{i}\left|b_{ij}\right|{\text{𝓁}}_{j}^{\text{2}}z_{j}^{\text{2}}\left(t-\tau_{ij}\right)\right)$$

$$\;=\sum_{i=\text{1}}^{n}\sum_{j=\text{1}}^{n}\left(\frac{\text{1}}{\varepsilon_{\text{2}}}\in_{i}\left|b_{ij}\right|{\text{𝓁}}_{i}^{\text{2}}z_{i}^{\text{2}}(t)+\varepsilon_{\text{2}}\;\;\in_{j}\left|b_{ji}\right|{\text{𝓁}}_{i}^{\text{2}}z_{i}^{\text{2}}(t-\tau_{ji})\right)$$

$$\text{2}\sum_{i=\text{1}}^{n}\sum_{j=}^{n}\frac{\in_{i}{\text{𝓁}}_{i}}{\alpha_{i}\left(z_{i}(t)\right)}e_{ij}z_{i}(t){\dot{z}}_{j}\left(t-\zeta_{ij}\right)\leq \sum_{i=\text{1}}^{n}\sum_{j=}^{n}\frac{\text{2}\left|e_{ij}\right|\in_{i}{\text{𝓁}}_{i}}{\alpha_{i}\left(z_{i}(t)\right)}\left|z_{i}(t)\right|\left|{\dot{z}}_{j}\left(t-\zeta_{ij}\right)\right|$$

$$\;\leq \text{2}\sum_{i=\text{1}}^{n}\sum_{j=\text{1}}^{n}\frac{\in_{i}{\text{𝓁}}_{i}}{v_{i}}\left|e_{ij}\right|\left|z_{i}(t)\right|\left|{\dot{z}}_{j}\left(t-\zeta_{ij}\right)\right|$$

$$\;\leq \sum_{i=\text{1}}^{n}\sum_{j=\text{1}}^{n}\left(\frac{\text{1}}{\varepsilon_{\text{3}}}\frac{\in_{i}}{v_{i}}{\text{𝓁}}_{i}^{\text{2}}\left|e_{ij}\right|z_{i}^{\text{2}}(t)+\varepsilon_{\text{3}}\frac{\in_{i}}{v_{i}}e_{ij}{\dot{z}}_{j}\left(t-\zeta_{ij}\right)\right)$$

$$\;=\sum_{i=\text{1}}^{n}\sum_{j=\text{1}}^{n}\left(\frac{\text{1}}{\varepsilon_{\text{3}}}\frac{\in_{i}}{v_{i}}{\text{𝓁}}_{i}^{\text{2}}\left|e_{ij}\right|z_{i}^{\text{2}}(t)+\varepsilon_{\text{3}}\frac{\in_{j}}{v_{j}}e_{ji}{\dot{z}}_{i}\left(t-\zeta_{ji}\right)\right)$$ (20)

$$\;\text{2}\sum_{i=\text{1}}^{n}\sum_{j=\text{1}}^{n}\in_{i}a_{ij}{\dot{z}}_{i}(t)g_{j}\left(z_{j}(t)\right)\leq \text{2}\sum_{i=\text{1}}^{n}\sum_{j=\text{1}}^{n}\in_{i}\left|a_{ij}\right||{\dot{z}}_{i}(t)|\left|g_{j}\left(z_{j}(t)\right)\right|$$

$$\;\leq \sum_{i=\text{1}}^{n}\sum_{j=\text{1}}^{n}\left(\frac{\text{1}}{\varepsilon_{\text{1}}}\in_{i}\left|a_{ij}\right|{\dot{z}}_{i}^{\text{2}}(t)+\varepsilon_{\text{1}}\;\;\in_{i}\left|a_{ij}\right|g_{j}^{\text{2}}\left(z_{j}(t)\right)\right)$$

$$\;\leq \sum_{i=\text{1}}^{n}\sum_{j=\text{1}}^{n}\left(\frac{\text{1}}{\varepsilon_{\text{1}}}\in_{i}\left|a_{ij}\right|{\dot{z}}_{i}^{\text{2}}(t)+\varepsilon_{\text{1}}\;\;\in_{i}\left|a_{ij}\right|{\text{𝓁}}_{j}^{\text{2}}z_{j}^{\text{2}}(t)\right)\;$$

$$\;=\sum_{i=\text{1}}^{n}\sum_{j=\text{1}}^{n}\left(\frac{\text{1}}{\varepsilon_{\text{1}}}\in_{i}\left|a_{ij}\right|{\dot{z}}_{i}^{\text{2}}(t)+\varepsilon_{\text{1}}\;\;\in_{j}\left|a_{ji}\right|{\text{𝓁}}_{i}^{\text{2}}z_{i}^{\text{2}}(t)\right)$$ (21)

$$\;\text{2}\sum_{i=\text{1}}^{n}\sum_{j=\text{1}}^{n}\in_{i}b_{ij}{\dot{z}}_{i}(t)g_{j}\left(z_{j}\left(t-\tau_{ij}\right)\right)\leq \text{2}\sum_{i=\text{1}}^{n}\sum_{j=\text{1}}^{n}\in_{i}\left|b_{ij}\right||{\dot{z}}_{i}(t)|\left|g_{j}\left(z_{j}\left(t-\tau_{ij}\right)\right)\right|$$

$$\;\leq \sum_{i=\text{1}}^{n}\sum_{j=\text{1}}^{n}\left(\frac{\text{1}}{\varepsilon_{\text{2}}}\in_{i}\left|b_{ij}\right|{\dot{z}}_{i}^{\text{2}}(t)+\varepsilon_{\text{2}}\;\;\in_{i}\left|b_{ij}\right|g_{j}^{\text{2}}\left(z_{j}\left(t-\tau_{ij}\right)\right)\right)$$

$$\;\leq \sum_{i=\text{1}}^{n}\sum_{j=\text{1}}^{n}\left(\frac{\text{1}}{\varepsilon_{\text{2}}}\in_{i}\left|b_{ij}\right|{\dot{z}}_{i}^{\text{2}}(t)+\varepsilon_{\text{2}}\;\;\in_{i}\left|b_{ij}\right|{\text{𝓁}}_{j}^{\text{2}}z_{j}^{\text{2}}\left(t-\tau_{ij}\right)\right)$$

$$\;=\sum_{i=\text{1}}^{n}\sum_{j=\text{1}}^{n}\left(\frac{\text{1}}{\varepsilon_{\text{2}}}\in_{i}\left|b_{ij}\right|{\dot{z}}_{i}^{\text{2}}(t)+\varepsilon_{\text{2}}\;\;\in_{j}\left|b_{ji}\right|{\text{𝓁}}_{i}^{\text{2}}z_{i}^{\text{2}}(t-\tau_{ji})\right)$$ (22)

and


$$\text{2}\sum_{i=\text{1}}^{n}\sum_{j=1}^{n}\frac{\in_{i}}{\alpha_{i}\left(z_{i}(t)\right)}e_{ij}{\dot{z}}_{i}(t){\dot{z}}_{j}\left(t-\zeta_{ij}\right)\leq \text{2}\sum_{i=\text{1}}^{n}\sum_{j=\text{1}}^{n}\frac{\in_{i}}{\alpha_{i}\left(z_{i}(t)\right)}\left|e_{ij}\right||{\dot{z}}_{i}(t)|\left|{\dot{z}}_{j}\left(t-\zeta_{ij}\right)\right|$$ (23)

$$\;\;\;\;\;\;\;\;\;\;\;\;\;\;\;\;\;\;\;\;\;\;\;\;\;\;\;\;\leq \text{2}\sum_{i=\text{1}}^{n}\sum_{j=1}^{n}\frac{\in_{i}}{v_{i}}\left|e_{ij}\right||{\dot{z}}_{i}(t)|\left|{\dot{z}}_{j}\left(t-\zeta_{ij}\right)\right|$$

$$\;\leq \sum_{i=\text{1}}^{n}\sum_{j=\text{1}}^{n}\left(\frac{\text{1}}{\varepsilon_{\text{3}}}\frac{\in_{i}}{v_{i}}\left|e_{ij}\right|{\dot{z}}_{i}^{\text{2}}(t)+\varepsilon_{\text{3}}\frac{\in_{i}}{v_{i}}\left|e_{ij}\right|{\dot{z}}_{j}^{\text{2}}\left(t-\zeta_{ij}\right)\right)$$

$$\;=\sum_{i=\text{1}}^{n}\sum_{j=\text{1}}^{n}\left(\frac{\text{1}}{\varepsilon_{\text{3}}}\frac{\in_{i}}{v_{i}}\left|e_{ij}\right|{\dot{z}}_{i}^{\text{2}}(t)+\varepsilon_{\text{3}}\frac{\in_{j}}{v_{j}}\left|e_{ji}\right|{\dot{z}}_{i}^{\text{2}}\left(t-\zeta_{ji}\right)\right)$$

Based on (18)-(23), (14) and (17) will respectively lead to


$$\xi {\dot{V}}_{\text{1}}(t)\leq -\text{2}\xi \sum_{i=\text{1}}^{n}\in_{i}\;{\text{𝓁}}_{i}\gamma_{i}z_{i}^{\text{2}}(t)+\frac{\xi }{\varepsilon_{\text{1}}}\sum_{i=\text{1}}^{n}\sum_{j=\text{1}}^{n}\in_{i}\;\left|a_{ij}\right|{\text{𝓁}}_{i}^{\text{2}}z_{i}^{\text{2}}(t)+\xi \varepsilon_{\text{1}}\sum_{i=\text{1}}^{n}\sum_{j=\text{1}}^{n}\in_{j}\left|a_{ji}\right|{\text{𝓁}}_{i}^{\text{2}}z_{i}^{\text{2}}(t)$$ (24)

$$\;+\frac{\xi }{\varepsilon_{\text{2}}}\sum_{i=\text{1}}^{n}\sum_{j=\text{1}}^{n}\in_{i}\;\left|b_{ij}\right|\;{\text{𝓁}}_{i}^{\text{2}}z_{i}^{\text{2}}(t)+\xi \varepsilon_{\text{2}}\sum_{i=\text{1}}^{n}\sum_{j=\text{1}}^{n}\;\in_{j}\;\left|b_{ji}\right|\;{\text{𝓁}}_{i}^{\text{2}}z_{i}^{\text{2}}\left(t-\tau_{ji}\right)\;$$

$$\;+\frac{\xi }{\varepsilon_{\text{3}}}\sum_{i=\text{1}}^{n}\sum_{j=\text{1}}^{n}\frac{\in_{i}}{\upsilon_{i}}{\text{𝓁}}_{i}^{\text{2}}\left|e_{ij}\right|z_{i}^{\text{2}}(t)+\xi \varepsilon_{\text{3}}\sum_{i=\text{1}}^{n}\sum_{j=\text{1}}^{n}\frac{\in_{j}}{\upsilon_{j}}e_{ji}{\dot{z}}_{i}\left(t-\zeta_{ji}\right)$$

and


$$\begin{array}{cc} (\text{1}-\xi ){\dot{V}}_{\text{2}}(t)\leq & \sum_{i=\text{1}}^{n}\left(-\text{2}(\text{1}-\xi )\frac{q_{i}}{\phi_{i}}{\dot{z}}_{i}^{\text{2}}(t)+\sum_{j=\text{1}}^{n}\left(\frac{\left(\text{1}-\xi \right)}{\varepsilon_{\text{1}}}q_{i}\left|a_{ij}\right|{\dot{z}}_{i}^{\text{2}}(t)+(\text{1}-\xi )\varepsilon_{\text{1}}q_{j}\left|a_{ji}\right|{\text{𝓁}}_{i}^{\text{2}}z_{i}^{\text{2}}(t)\right)\right)\\ & +\sum_{i=\text{1}}^{n}\sum_{j=\text{1}}^{n}\left(\frac{\left(\text{1}-\xi \right)}{\varepsilon_{\text{2}}}\in_{i}\left|b_{ij}\right|{\dot{z}}_{i}^{\text{2}}(t)+(\text{1}-\xi )\varepsilon_{\text{2}}q_{j}\left|b_{ji}\right|{\text{𝓁}}_{i}^{\text{2}}z_{i}^{\text{2}}\left(t-\tau_{ji}\right)\right)\\ & +\sum_{i=\text{1}}^{n}\sum_{j=\text{1}}^{n}\left(\frac{\left(\text{1}-\xi \right)}{\varepsilon_{\text{3}}}\frac{\in_{i}}{\upsilon_{i}}\left|e_{ij}\right|{\dot{z}}_{i}^{\text{2}}(t)+(\text{1}-\xi )\varepsilon_{\text{3}}\frac{q_{j}}{\upsilon_{j}}\left|e_{ji}\right|{\dot{z}}_{i}^{\text{2}}\left(t-\zeta_{ji}\right)\right) \end{array}$$ (25)

Combining (24) and (25) results in


$$\xi {\dot{V}}_{\text{1}}(t)+(\text{1}-\xi ){\dot{V}}_{\text{2}}(t)$$

$$\;\leq \sum_{i=\text{1}}^{n}\left(-\text{2}\xi \;\;\in_{i}{\text{𝓁}}_{i}\gamma_{i}z_{i}^{\text{2}}(t)+\sum_{j=\text{1}}^{n}\left(\frac{\xi }{\varepsilon_{\text{1}}}\in_{i}\left|a_{ij}\right|{\text{𝓁}}_{i}^{\text{2}}z_{i}^{\text{2}}(t)+\varepsilon_{\text{1}}\in_{j}\left|a_{ji}\right|{\text{𝓁}}_{i}^{\text{2}}z_{i}^{\text{2}}(t)\right)\right)\;$$

$$\;+\sum_{i=\text{1}}^{n}\sum_{j=\text{1}}^{n}\left(\frac{\xi }{\varepsilon_{\text{2}}}\in_{i}\left|b_{ij}\right|{\text{𝓁}}_{i}^{\text{2}}z_{i}^{\text{2}}(t)+\frac{\xi }{\varepsilon_{\text{3}}}\frac{\in_{i}}{\upsilon_{i}}{\text{𝓁}}_{i}^{\text{2}}\left|e_{ij}\right|z_{i}^{\text{2}}(t)+\varepsilon_{\text{2}}\in_{j}\left|b_{ji}\right|{\text{𝓁}}_{i}^{\text{2}}z_{i}^{\text{2}}\left(t-\tau_{ji}\right)\right)$$

$$\;+\sum_{i=\text{1}}^{n}\left(-\text{2}(\text{1}-\xi )\frac{\in_{i}}{\phi_{i}}{\dot{z}}_{i}^{\text{2}}(t)+\sum_{j=\text{1}}^{n}\left(\frac{\left(\text{1}-\xi \right)}{\varepsilon_{\text{1}}}\in_{i}\left|a_{ij}\right|{\dot{z}}_{i}^{\text{2}}(t)+\in_{i}\left|b_{ij}\right|{\text{𝓁}}_{i}^{\text{2}}{\dot{z}}_{i}^{\text{2}}(t)\right)\right)$$

$$\;+\sum_{i=\text{1}}^{n}\sum_{j=\text{1}}^{n}\left(\frac{\left(\text{1}-\xi \right)}{\varepsilon_{\text{3}}}\frac{\in_{i}}{\upsilon_{i}}\left|e_{ij}\right|{\dot{z}}_{i}^{\text{2}}(t)+\varepsilon_{\text{3}}\frac{\in_{j}}{\upsilon_{j}}\left|e_{ji}\right|{\dot{z}}_{i}^{\text{2}}\left(t-\zeta_{ji}\right)\right)$$ (26)

Finally, following inequality is calculated for ${\dot{V}}_{\text{3}}(t)$:


$${\dot{V}}_{\text{3}}(t)=\varepsilon_{\text{2}}\sum_{i=\text{1}}^{n}\sum_{j=\text{1}}^{n}\left(\in_{j}\left|b_{ji}\right|{\text{𝓁}}_{i}^{\text{2}}z_{i}^{\text{2}}(t)-\in_{j}\left|b_{ji}\right|{\text{𝓁}}_{i}^{\text{2}}z_{i}^{\text{2}}\left(t-\tau_{ji}\right)\right)$$ (27)

$$\;+\varepsilon_{\text{3}}\sum_{i=\text{1}}^{n}\sum_{j=\text{1}}^{n}\left(\frac{\in_{j}}{\upsilon_{j}}e_{ji}{\dot{z}}_{i}^{\text{2}}(t)-\frac{\in_{j}}{\upsilon_{j}}e_{ji}{\dot{z}}_{i}^{\text{2}}\left(t-\zeta_{ji}\right)\right)\;$$

$$\;+\sum_{i=\text{1}}^{n}\sum_{j=\text{1}}^{n}\left(\frac{p}{n}\left(z_{j}^{\text{2}}(t)-z_{j}^{\text{2}}\left(t-\tau_{ij}\right)\right)+\frac{q}{n}\left({\dot{z}}_{j}^{\text{2}}(t)-{\dot{z}}_{j}^{\text{2}}\left(t-\zeta_{ij}\right)\right)\right)$$

$$\;\leq \varepsilon_{\text{2}}\sum_{i=\text{1}}^{n}\sum_{j=\text{1}}^{n}\left(\in_{j}\left|b_{ji}\right|{\text{𝓁}}_{i}^{\text{2}}z_{i}^{\text{2}}(t)-\in_{j}\left|b_{ji}\right|{\text{𝓁}}_{i}^{\text{2}}z_{i}^{\text{2}}\left(t-\tau_{ji}\right)\right)$$

$$\;+\varepsilon_{\text{3}}\sum_{i=\text{1}}^{n}\sum_{j=\text{1}}^{n}\left(\frac{\in_{j}}{\upsilon_{j}}e_{ji}{\dot{z}}_{i}^{\text{2}}(t)-\frac{\in_{j}}{\upsilon_{j}}e_{ji}{\dot{z}}_{i}^{\text{2}}\left(t-\zeta_{ji}\right)\right)+\sum_{i=\text{1}}^{n}\sum_{j=\text{1}}^{n}\left(\frac{p}{n}z_{j}^{\text{2}}(t)+\frac{q}{n}{\dot{z}}_{j}^{\text{2}}(t)\right)$$

Combining equation (27) with (26)


$$\dot{V}(t)=\xi {\dot{V}}_{\text{1}}(t)+(\text{1}-\xi ){\dot{V}}_{\text{2}}(t)+{\dot{V}}_{\text{3}}(t)\;$$

$$\;\leq -\text{2}\xi \sum_{i=\text{1}}^{n}\in_{i}{\text{𝓁}}_{i}\gamma_{i}z_{i}^{\text{2}}(t)+\frac{\xi }{\varepsilon_{\text{1}}}\sum_{i=\text{1}}^{n}\sum_{j=\text{1}}^{n}\in_{i}\left|a_{ij}\right|{\text{𝓁}}_{i}^{\text{2}}z_{i}^{\text{2}}(t)+\varepsilon_{\text{1}}\sum_{i=\text{1}}^{n}\sum_{j=\text{1}}^{n}\in_{j}\left|a_{ji}\right|{\text{𝓁}}_{i}^{\text{2}}z_{i}^{\text{2}}(t)\;$$

$$\;+\frac{\xi }{\varepsilon_{\text{2}}}\sum_{i=\text{1}}^{n}\sum_{j=\text{1}}^{n}\in_{i}\left|b_{ij}\right|{\text{𝓁}}_{i}^{\text{2}}z_{i}^{\text{2}}(t)\;+\varepsilon_{\text{2}}\sum_{i=\text{1}}^{n}\sum_{j=\text{1}}^{n}\in_{j}\left|b_{ji}\right|{\text{𝓁}}_{i}^{\text{2}}z_{i}^{\text{2}}(t)+\frac{\xi }{\varepsilon_{\text{3}}}\sum_{i=\text{1}}^{n}\sum_{j=\text{1}}^{n}\frac{\in_{i}}{\upsilon_{i}}{\text{𝓁}}_{i}^{\text{2}}\left|e_{ij}\right|z_{i}^{\text{2}}(t)$$

$$\;-\text{2}(\text{1}-\xi )\sum_{i=\text{1}}^{n}\frac{\in_{i}}{\phi_{i}}{\dot{z}}_{i}^{\text{2}}(t)+\frac{\left(\text{1}-\xi \right)}{\varepsilon_{\text{1}}}\sum_{i=\text{1}}^{n}\sum_{j=\text{1}}^{n}\in_{i}\left|a_{ij}\right|{\dot{z}}_{i}^{\text{2}}(t)+\frac{\left(\text{1}-\xi \right)}{\varepsilon_{\text{2}}}\sum_{i=\text{1}}^{n}\sum_{j=\text{1}}^{n}\in_{i}\left|b_{ij}\right|{\dot{z}}_{i}^{\text{2}}(t)$$

$$\;+\frac{\left(\text{1}-\xi \right)}{\varepsilon_{\text{3}}}\sum_{i=\text{1}}^{n}\sum_{j=\text{1}}^{n}\frac{\in_{i}}{\upsilon_{i}}\left|e_{ij}\right|{\dot{z}}_{i}^{\text{2}}(t)+\varepsilon_{\text{3}}\sum_{i=\text{1}}^{n}\sum_{j=\text{1}}^{n}\frac{\in_{i}}{\upsilon_{j}}\left|e_{ji}\right|{\dot{z}}_{i}^{\text{2}}(t)+\sum_{i=\text{1}}^{n}\sum_{j=\text{1}}^{n}\left(\frac{p}{n}z_{j}^{\text{2}}(t)+\frac{q}{n}{\dot{z}}_{j}^{\text{2}}(t)\right)$$

$$=-\sum_{i=\text{1}}^{n}{\text{𝓁}}_{i}^{\text{2}}\left(\text{2}\xi \in_{i}\frac{\gamma_{i}}{{\text{𝓁}}_{i}}\;-\sum_{j=\text{1}}^{n}\left(\frac{\xi }{\varepsilon_{\text{1}}}\in_{i}\left|a_{ij}\right|+{\varepsilon_{\text{1}}\in }_{j}\left|a_{ji}\right|\right)-\sum_{j=\text{1}}^{n}\left(\frac{\xi }{\varepsilon_{\text{2}}}\in_{i}\left|b_{ij}\right|+\varepsilon_{\text{2}}\in_{j}\left|b_{ji}\right|\right)-\frac{\xi }{\varepsilon_{\text{3}}}\sum_{j=\text{1}}^{n}\frac{\in_{i}}{\upsilon_{i}}e_{ij}\right)z_{i}^{\text{2}}(t)$$

$$\begin{array}{cc} \; & -\sum_{i=\text{1}}^{n}\left(\text{2}(\text{1}-\xi )\frac{\in_{i}}{\phi_{i}}-\frac{\left(\text{1}-\xi \right)}{\varepsilon_{\text{1}}}\sum_{j=\text{1}}^{n}\in_{i}\left|a_{ij}\right|-\frac{\left(\text{1}-\xi \right)}{\varepsilon_{\text{2}}}\sum_{j=\text{1}}^{n}\in_{i}\left|b_{ij}\right|-\frac{\left(\text{1}-\xi \right)}{\varepsilon_{\text{3}}}\sum_{j=\text{1}}^{n}\frac{\in_{i}}{\upsilon_{i}}\left|e_{ij}\right|-\varepsilon_{\text{3}}\sum_{j=\text{1}}^{n}\frac{\in_{j}}{\upsilon_{j}}\left|e_{ji}\right|\right){\dot{z}}_{i}^{\text{2}}(t)\\ & +\sum_{i=\text{1}}^{n}\sum_{j=\text{1}}^{n}\left(\frac{p}{n}z_{j}^{\text{2}}(t)+\frac{q}{n}{\dot{z}}_{j}^{\text{2}}(t)\right) \end{array}$$

$$=-\sum_{i=\text{1}}^{n}{\text{𝓁}}_{i}^{\text{2}}\delta_{i}z_{i}^{\text{2}}(t)-\sum_{i=\text{1}}^{n}\kappa_{i}{\dot{z}}_{i}^{\text{2}}(t)+p\sum_{i=\text{1}}^{n}z_{i}^{\text{2}}(t)+q\sum_{i=\text{1}}^{n}{\dot{z}}_{i}^{\text{2}}(t)$$

$$\leq -{\text{𝓁}}_{m}^{\text{2}}\delta_{m}\sum_{i=\text{1}}^{n}z_{i}^{\text{2}}(t)-\kappa_{m}\sum_{i=\text{1}}^{n}{\dot{z}}_{i}^{\text{2}}(t)+p\sum_{i=\text{1}}^{n}z_{i}^{\text{2}}(t)+q\sum_{i=\text{1}}^{n}{\dot{z}}_{i}^{\text{2}}(t)$$

$$\;=-\left({\text{𝓁}}_{m}^{\text{2}}\delta_{m}-p\right){\left\|z(t)\right\|}_{\text{2}}^{\text{2}}-(\kappa_{m}-q){\left\|\dot{z}(t)\right\|}_{\text{2}}^{\text{2}}$$ (28)

in which ${\text{𝓁}}_{m}=\text{min}\left({\text{𝓁}}_{i}\right),\;\delta_{m}=\text{min}\left(\delta_{i}\right)$ and $\kappa_{m}=\text{min}(\kappa_{i})$. In (28), the conditions $p<{\text{𝓁}}_{m}^{\text{2}}\delta_{m}$ and $q<\kappa_{m}$ assure that $\dot{V}(t)<\text{0}$ hold if z(t)≠0. Additionally, it can be examined that $\dot{V}(t)<\text{0}$ is also satisfied under the condition that $\dot{z}(t)\neq \text{0}$. Moreover, one can also derive that if $z(t)=\dot{z}(t)=\text{0}$, then ${\dot{V}}_{\text{1}}(t)=\text{0}$ and ${\dot{V}}_{\text{2}}(t)=\text{0}$, which signifies that $\dot{V}(t)={\dot{V}}_{\text{3}}(t)$. Hence, equation (27) directly yields the inequality.


$$\dot{V}(t)=\sum_{i=\text{1}}^{n}\sum_{j=\text{1}}^{n}\left(-\varepsilon_{\text{2}}\in_{j}\left|b_{ji}\right|{\text{𝓁}}_{i}^{\text{2}}z_{i}^{\text{2}}\left(t-\tau_{ji}\right)-\varepsilon_{\text{3}}\frac{\in_{j}}{v_{j}}e_{ji}{\dot{z}}_{i}^{\text{2}}\left(t-\zeta_{ji}\right)\right)$$

$$\;-\frac{\text{1}}{n}\sum_{i=\text{1}}^{n}\sum_{j=\text{1}}^{n}\left(pz_{j}^{\text{2}}\left(t-\tau_{ij}\right)+q{\dot{z}}_{j}^{\text{2}}\left(t-\zeta_{ij}\right)\right)$$

$$\;\leq -\frac{\text{1}}{n}\sum_{i=\text{1}}^{n}\sum_{j=\text{1}}^{n}\left(pz_{j}^{\text{2}}\left(t-\tau_{ij}\right)+q{\dot{z}}_{j}^{\text{2}}\left(t-\zeta_{ij}\right)\right)$$ (29)

It is obvious from inequality (29) that if the states satisfy the conditions $z_{j}(t-\tau_{ij})\neq \text{0}$ for some arbitrary chosen indices i and j, in which case, (29) directly guarantees that $\dot{V}(t)<\text{0}$. Additionaly, if ${\dot{z}}_{j}(t-\tau_{ij})\neq \text{0}$ for some arbitrary chosen indices i and j, then (29) also directly guarantees that $\dot{V}(t)<\text{0}$. We can also observe that, in all cases of $z_{j}(t-\tau_{ij})$ as well as ${\dot{z}}_{j}(t-\tau_{ij})=\text{0},\;\forall i,\;j$ together with the condition $\dot{z}(t)=z(t)=\text{0}$, it follows that ${\dot{V}}_{\text{1}}(t)={\dot{V}}_{\text{2}}(t)={\dot{V}}_{\text{3}}(t)=\text{0},$ assuring the condition of $\dot{V}(t)=\text{0}$. For the sake of ensuring exact global convergence and stability properties of origin, we require to control whether V(t) is radially unbounded. One may write:


$$V(t)\geq \sum_{i=\text{1}}^{n}\text{2}\in_{i}\int_{\text{0}}^{z_{i}(t)}\beta_{i}(s)ds\geq \text{2}\sum_{i=\text{1}}^{n}\in_{m}\int_{\text{0}}^{z_{i}(t)}\gamma_{i}(s)sds$$

$$\;\geq \text{2}\in_{m}\;\;\gamma_{m}\sum_{i=\text{1}}^{n}\int_{\text{0}}^{z_{i}(t)}sds=\in_{m}\;\;\gamma_{m}{\left\|z(t)\right\|}_{\text{2}}^{\text{2}}$$ (30)

From (30), we may realise that V(t)→∞ holds if ‖z(t)‖→∞, proving that the positive real valued V(t) is certainly radially unbounded. Q.E.D.

Some useful particular cases of results of Theorem 1 are stated below. For $\varepsilon_{\text{1}}=\varepsilon_{\text{2}}=\varepsilon_{\text{3}}=\sqrt{\xi }$, Theorem 1 directly yields the following stability criteria:

### 2.2. Corollary 1

Let neural network (1) be governed by system functions that satisfy conditions $H_{\text{1}}$, $H_{\text{2}}$ and $H_{\text{3}}$. Under these conditions, the neural network (1) is globally asymptotically stable if there exist some positive real constants $\in_{i}>\text{0}$ satisfying the following algebraic criteria:


$${\hat{\delta }}_{i}=\text{2}\sqrt{\xi }\frac{\gamma_{i}}{{\text{𝓁}}_{i}}-\sum_{j=\text{1}}^{n}\left(\in_{i}\left(\left|a_{ij}\right|+\left|b_{ij}\right|\right)+\in_{j}\left(\left|a_{ji}\right|+\left|b_{ji}\right|\right)\right)\;-\frac{\in_{i}}{\upsilon_{i}}\sum_{j=\text{1}}^{n}\left|e_{ij}\right|>\text{0},\;\forall i$$ (31)

and


$${\hat{\kappa }}_{i}=\text{2}(\text{1}-\xi )\frac{\in_{i}}{\phi_{i}}-\frac{\text{1}-\xi }{\sqrt{\xi }}\in_{i}\left(\sum_{j=\text{1}}^{n}\left(\left|a_{ij}\right|+\left|b_{ij}\right|+\frac{\text{1}}{\upsilon_{i}}\left|e_{ij}\right|\right)\right)-\sqrt{\xi }\sum_{j=\text{1}}^{n}\frac{\in_{j}}{\upsilon_{j}}\left|e_{ji}\right|>\text{0},\;\forall i$$ (32)

where 0<ξ<1. For $\in_{i}\;=\text{1},\;\forall i,\;r=min\left(\frac{\gamma_{i}}{{\text{𝓁}}_{i}}\right),\;\upsilon_{m}=min\left(\upsilon_{i}\right),\;\phi_{M}=\text{max}\left(\phi_{i}\right)$, $\varepsilon_{\text{1}}=\sqrt{\xi }\frac{\sqrt{{\left\|A\right\|}_{\infty }}}{\sqrt{{\left\|A\right\|}_{\text{1}}}},\;\varepsilon_{\text{2}}=\sqrt{\xi }\frac{\sqrt{{\left\|B\right\|}_{\infty }}}{\sqrt{{\left\|B\right\|}_{\text{1}}}}$ and $\varepsilon_{\text{3}}=\sqrt{\xi }\frac{\sqrt{{\left\|E\right\|}_{\infty }}}{\sqrt{{\left\|E\right\|}_{\text{1}}}}$.

The following corollary results from Theorem 1.

### 2.3. Corollary 2

Let neural network (1) be governed by system functions that satisfy conditions $H_{\text{1}}$, $H_{\text{2}}$ and $H_{\text{3}}$. Under these conditions, the neural network (1) is globally asymptotically stable if the algebraic criteria given in the following form are satisfied,


$$\delta =\text{2}\sqrt{\xi }r-\text{2}\sqrt{{\left\|A\right\|}_{\infty }{\left\|A\right\|}_{\text{1}}}-\text{2}\sqrt{{\left\|B\right\|}_{\infty }{\left\|B\right\|}_{\text{1}}}-\frac{\text{1}}{\upsilon_{m}}\sqrt{{\left\|E\right\|}_{\infty }{\left\|E\right\|}_{\text{1}}}>\text{0}$$ (33)

and


$$\kappa =(\text{1}-\xi )\frac{\text{2}}{\phi_{M}}-\frac{\left(\text{1}-\xi \right)}{\sqrt{\xi }}\left(\sqrt{{\left\|A\right\|}_{\infty }{\left\|A\right\|}_{\text{1}}}+\sqrt{{\left\|B\right\|}_{\infty }{\left\|B\right\|}_{\text{1}}}\right)-\frac{\text{1}}{\sqrt{\xi }\upsilon_{m}}\sqrt{{\left\|E\right\|}_{\infty }{\left\|E\right\|}_{\text{1}}}>\text{0},$$ (34)

where 0<ξ<1.

When the results of Corollary 1 and Corollary 2 are compared, the following conclusions can be drawn: The result of Corollary 2 is a special case of Corollary 1. Corollary 1 imposes less restrictive constraint conditions on the network parameters of neural system (1) than those imposed by Corollary 2. However, it is more difficult to validate the conditions stated in Corollary 1 than the validation of the conditions stated in Corollary 2.

### 2.4. An example and comparisons

The aim of the current section will be the analysis of an example for the sake of comparisons between the results of this study and some existing stability conditions proposed in the literature. Firstly, we are required to restate some previous literature results.

#### 2.4.1. Theorem 2 [39].

Let neural network (1) be governed by system functions that satisfy conditions $H_{\text{1}}$, $H_{\text{2}}$ and $H_{\text{3}}$. Under these conditions, the neural network (1) is globally asymptotically stable if the algebraic criteria given in the following form are satisfied,


$$\rho_{i}=\frac{\gamma_{i}^{\text{2}}}{{\text{𝓁}}_{i}^{\text{2}}}-\sum_{j=\text{1}}^{n}\left|\sum_{k=\text{1}}^{n}a_{ki}a_{kj}\right|-\sum_{j=\text{1}}^{n}\sum_{k=\text{1}}^{n}\left(\left|a_{ji}\right|\left|b_{jk}\right|+\left|a_{ji}\right|\left|e_{jk}\right|+\left|a_{jk}\right|\left|b_{ji}\right|+\left|b_{ji}\right|\left|e_{jk}\right|+\left|b_{ji}\right|\left|b_{jk}\right|\right)>\text{0}$$ (35)

and


$$\varrho_{ij}=\frac{\text{1}}{n\phi_{j}^{\text{2}}}-\frac{\left|e_{ji}\right|}{\upsilon_{i}^{\text{2}}}\sum_{k=\text{1}}^{n}\left(\left|e_{jk}\right|+\left|a_{jk}\right|+\left|b_{jk}\right|\right)>\text{0},\;\forall i,j.$$ (36)

#### 2.4.2 Theorem 3 [40].

Let neural network (1) be governed by system functions that satisfy conditions $H_{\text{1}}$, $H_{\text{2}}$ and $H_{\text{3}}$. Under these conditions, the neural network (1) is globally asymptotically stable if the algebraic criteria given in the following form are satisfied,


$$\begin{array}{cc} v_{i}=\; & \text{2}\upsilon_{i}\gamma_{i}-\sum_{j=\text{1}}^{n}\left(\phi_{i}{\text{𝓁}}_{j}\left|a_{ij}\right|+\phi_{j}{\text{𝓁}}_{i}\left|a_{ji}\right|\right)-\sum_{j=\text{1}}^{n}\left(\phi_{i}{\text{𝓁}}_{j}\left|b_{ij}\right|+\phi_{j}{\text{𝓁}}_{i}\left|b_{ji}\right|\right)-\sum_{j=\text{1}}^{n}\left(\phi_{i}\psi_{i}\left|e_{ij}\right|+\phi_{j}\psi_{j}\left|e_{ji}\right|\right)\;\\ & \;\;-{\text{𝓁}}_{i}\sum_{j=\text{1}}^{n}\sum_{k=\text{1}}^{n}\left(\phi_{i}\left|a_{ki}\right|\left|e_{kj}\right|+\phi_{k}\left|b_{ki}\right|\left|e_{kj}\right|\right)-\left|e_{ji}\right|\sum_{j=\text{1}}^{n}\sum_{k=\text{1}}^{n}{\text{𝓁}}_{k}\left(\phi_{j}\left|a_{jk}\right|+\phi_{j}\left|b_{jk}\right|\right)>\text{0},\forall i \end{array}$$ (37)

and


$$e_{i}=\text{1}-\sum_{j=\text{1}}^{n}\left|e_{ji}\right|>\text{0},\;\forall i$$ (38)

#### 2.4.3 Theorem 4 [41].

Let neural network (1) be governed by system functions that satisfy conditions $H_{\text{1}}$, $H_{\text{2}}$ and $H_{\text{3}}$. Under these conditions, the neural network (1) is globally asymptotically stable if there exist real constants $k_{i}>\text{0}$ satisfying the following algebraic criteria:


$$m_{i}=k_{i}\upsilon_{i}\frac{\gamma_{i}}{{\text{𝓁}}_{i}}-\sum_{j=\text{1}}^{n}k_{j}\phi_{j}\left(\left|a_{ji}\right|+\left|b_{ji}\right|\right)>\text{0},\;\forall i$$ (39)

and


$${\hat{e}}_{i}=k_{i}-\sum_{j=\text{1}}^{n}k_{j}\left|e_{ji}\right|>\text{0},\;\forall i.$$ (40)

#### 2.4.4 Theorem 5 [42].

Let neural network (1) be governed by system functions that satisfy conditions $H_{\text{1}}$, $H_{\text{2}}$ and $H_{\text{3}}$. Under these conditions, the neural network (1) is globally asymptotically stable if there exist real constants $k_{i}>\text{0}$ satisfying the following algebraic criteria:


$$\sigma_{i}=k_{i}\frac{\gamma_{i}}{{\text{𝓁}}_{i}}-\sum_{j=\text{1}}^{n}k_{j}\left(\left|a_{ji}\right|+\left|b_{ji}\right|\right)>\text{0},\;\forall i$$ (41)

and


$${\tilde{e}}_{i}=\sum_{j=\text{1}}^{n}\left|e_{ji}\right|<\frac{\text{1}}{\sigma },\;\forall i,\;\sigma \frac{k_{j}}{\phi_{j}}-\frac{k_{i}}{\upsilon_{i}}\geq \text{0},\;\forall i,\;j,\;\sigma \geq \frac{k_{M}\phi_{M}}{k_{m}\upsilon_{m}}\geq \text{1}$$ (42)

#### 2.4.5 Theorem 6 [43].

Let neural network (1) be governed by system functions that satisfy conditions $H_{\text{1}}$, $H_{\text{2}}$ and $H_{\text{3}}$. Under these conditions, the neural network (1) is globally asymptotically stable if there exist real constants $\Omega_{i}>\text{0},\;(i=\text{1},\;\text{2},\;\text{3},\;\text{4},\;\text{5})$ satisfying the following algebraic criteria:


$$\begin{array}{cc} \pi_{i}=\; & \text{2}\upsilon_{i}\gamma_{i}-\phi_{i}^{\text{2}}\left(\Omega_{\text{1}}+\Omega_{\text{2}}\right)-\frac{\text{1}}{\Omega_{\text{3}}}\psi_{i}^{\text{2}}-\left(\Omega_{\text{3}}+\Omega_{\text{4}}+\Omega_{\text{5}}\right)\sum_{j=\text{1}}^{n}\sum_{k=\text{1}}^{n}\phi_{j}^{\text{2}}\left|e_{ji}e_{jk}\right|\\ & -\left(\frac{\text{1}}{\Omega_{\text{1}}}+\frac{\text{1}}{\Omega_{\text{4}}}\right)\sum_{j=\text{1}}^{n}\left|\sum_{k=\text{1}}^{n}a_{ki}a_{kj}\right|{\text{𝓁}}_{i}^{\text{2}}-\left(\frac{\text{1}}{\Omega_{\text{2}}}+\frac{\text{1}}{\Omega_{\text{5}}}\right)\sum_{j=\text{1}}^{n}\sum_{k=\text{1}}^{n}\left|b_{ki}b_{kj}\right|{\text{𝓁}}_{i}^{\text{2}}>\text{0},\forall i \end{array}$$ (43)

The following example will lead us to compare the proposed results with some of the existing stability conditions.

#### 2.4.6 Example.

Consider system (1) that has system parameters:

$A=a\left[\begin{array}{c} \begin{array}{c} \begin{array}{c} \begin{array}{cc} \;\;\;\;\;\;\;\;\text{1} & \begin{array}{cc} \text{1} & \begin{array}{cc} \text{1} & \text{1} \end{array} \end{array} \end{array}\\ \begin{array}{ccc} \;\;\;\;\;\;\;\;\text{1} & \text{1} & \begin{array}{cc} \text{1} & \text{1} \end{array} \end{array} \end{array}\\ \;\;\;\;\;\begin{array}{ccc} \text{1} & \text{1} & \begin{array}{cc} \text{1} & \text{1} \end{array} \end{array} \end{array}\\ \;\;\begin{array}{ccc} \text{1} & \text{1} & \begin{array}{cc} \text{1} & \text{1} \end{array} \end{array} \end{array}\;\;\;\;\;\;\right]$, $B=b\left[\begin{array}{c} \begin{array}{c} \begin{array}{c} \begin{array}{cc} \;\;\;\;\;\;\;\;\;\text{1} & \begin{array}{cc} \text{1} & \begin{array}{cc} \text{1} & \text{1} \end{array} \end{array} \end{array}\\ \;\;\;\;\;\;\;\;\;\begin{array}{ccc} \text{1} & \text{1} & \begin{array}{cc} \text{1} & \text{1} \end{array} \end{array} \end{array}\\ \;\;\;\;\;\;\begin{array}{ccc} \text{1} & \text{1} & \begin{array}{cc} \text{1} & \text{1} \end{array} \end{array} \end{array}\\ \;\;\;\begin{array}{ccc} \text{1} & \text{1} & \begin{array}{cc} \text{1} & \text{1} \end{array} \end{array} \end{array}\;\;\;\;\;\;\;\right]$, $E=e\left[\begin{array}{c} \begin{array}{c} \begin{array}{c} \;\;\;\;\;\;\;\;\begin{array}{cc} \text{1} & \begin{array}{cc} \text{1} & \begin{array}{cc} \text{0} & \text{4} \end{array} \end{array} \end{array}\\ \;\;\;\;\;\;\;\;\begin{array}{ccc} \text{1} & \text{0} & \begin{array}{cc} \text{1} & \text{4} \end{array} \end{array} \end{array}\\ \;\;\;\;\;\begin{array}{ccc} \text{0} & \text{1} & \begin{array}{cc} \text{1} & \text{4} \end{array} \end{array} \end{array}\\ \;\;\begin{array}{ccc} \text{1} & \text{1} & \begin{array}{cc} \text{0} & \text{4} \end{array} \end{array} \end{array}\;\;\;\;\;\;\;\right]$

a and b are real-valued positive constants, and $\upsilon_{m}=\text{1},\;\phi_{M}=\text{1},\;r=\text{1},\;e=\frac{\text{1}}{\text{15}},\;\psi_{\text{1}}=\psi_{\text{2}}=\psi_{\text{3}}=\psi_{\text{4}}=\sqrt{\text{6}}$,

Let $\xi =\frac{\text{1}}{\text{6}}$. Then, the conditions in Corollary 2 are calculated as:


$$\delta =\text{2}r\sqrt{\xi }-\text{8}a-\text{8}b-\text{4}\sqrt{\text{6}}e=\text{2}\left(\sqrt{\xi }-\text{4}a-\text{4}b-\text{2}\sqrt{\text{6}}e\right)=\text{2}\left(\frac{\text{1}}{\text{5}\sqrt{\text{6}}}-\text{4}a-\text{4}b\right)$$ (44)

and


$$\kappa =\frac{\text{5}}{\text{3}}-\text{24}e-\frac{\left(\text{1}-\xi \right)}{\sqrt{\xi }}(\text{4}a+\text{4}b)=\frac{\text{5}}{\text{3}}-\frac{\text{24}}{\text{15}}-\frac{\left(\text{1}-\xi \right)}{\sqrt{\xi }}(\text{4}a+\text{4}b)$$

$$\;=\frac{\text{1}}{\text{15}}-\frac{\left(\text{1}-\xi \right)}{\sqrt{\xi }}(\text{4}a+\text{4}b)=\frac{\text{1}}{\text{15}}-\frac{\text{5}}{\sqrt{\text{6}}}(\text{4}a+\text{4}b)$$ (45)

If $a+b<\frac{\text{1}}{\text{20}\sqrt{\text{6}}}$, then δ>0, and if $a+b<\frac{\text{1}}{\text{50}\sqrt{\text{6}}}$, then κ>0. Therefore, the condition $a+b<\frac{\text{1}}{\text{50}\sqrt{\text{6}}}$ ensures that the conditions stated in Corollary 2 hold.

For this example, let a=0,003, b=0,004. The state responses of the system (1) are illustrated using graphical representations respectively for the different network functions given in the following equations:


f(x)= d(x)=tanh(x), c(x)=sigmoid(x) and

f(x)=0,5tanh(x), d(x)=1−0,5cosx, c(x)=x

We also choose different amplified functions in the following forms for each neuron:

$f(x)=\text{0}.\text{0}\text{5}\text{tanh}(x),\;c(x)=\text{0}.\text{5}x,\;d_{\text{1}}(x)=\text{1}.\text{5}-\left|\text{tanh}(x)\right|,\;d_{\text{2}}(x)=\text{1}.\text{5}-\text{tanh}(x),$

$d_{\text{3}}(x)=\text{1}.\text{5}+\left|\text{tanh}(x)\right|,\;d_{\text{4}}(x)=\text{1}.\text{5}+\text{tanh}(x).$

The state responses of system (1) for the given network functions which are different from each other are shown in Fig 3.

**Fig 1:**
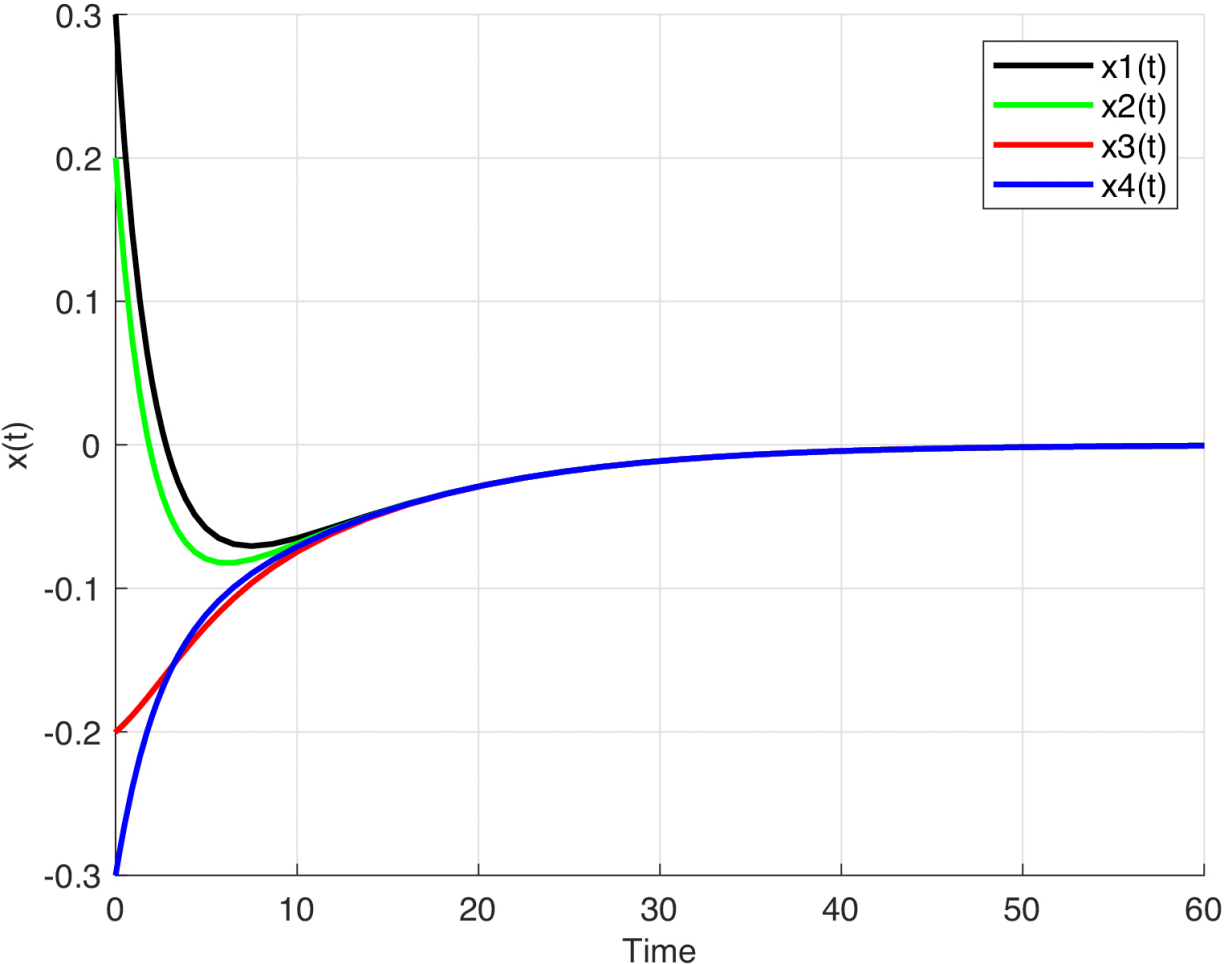
The time response of the system (1). The time response of the system (1) with f(x)= d(x)=tanh(x), c(x)=sigmoid(x).

**Fig 2:**
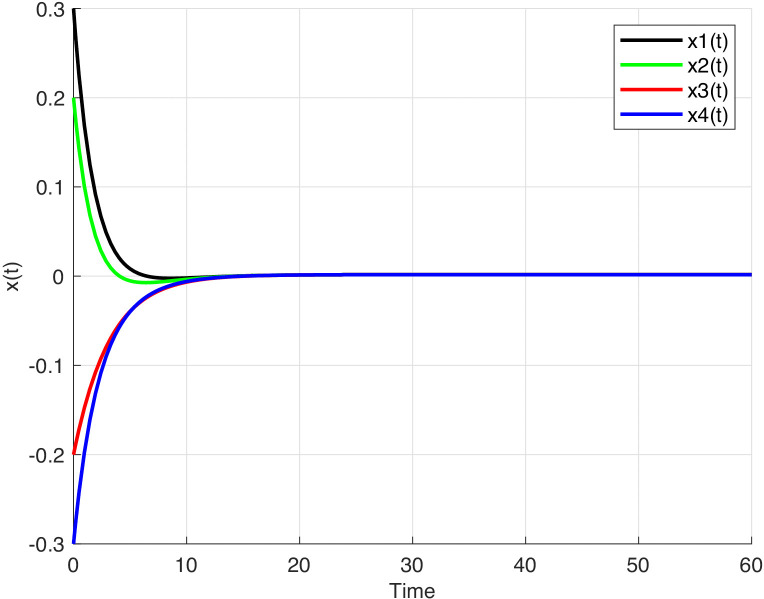
The time response of the system (1). The time response of the system (1) with f(x)=0.5tanh(x),  d(x)=1−0.5cosx, c(x)=x.

**Fig 3:**
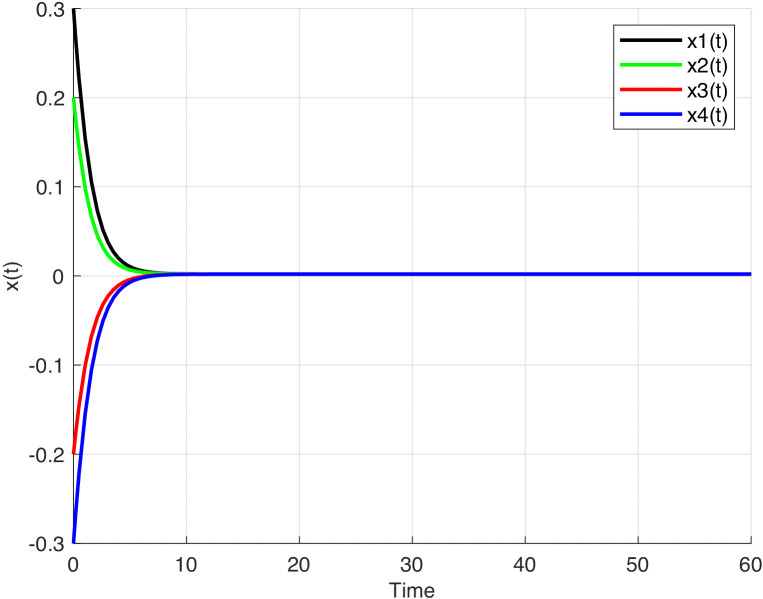
The time response of the system (1). The time response of the system (1) with $f(x)=\text{0}.\text{0}\text{5}\text{tanh}(x),c(x)=\text{0}.\text{5}x,d_{\text{1}}(x)=\text{1}.\text{5}-\left|\text{tanh}(x)\right|,$$d_{\text{2}}(x)=\text{1}.\text{5}-\text{tanh}(x),d_{\text{3}}(x)=\text{1}.\text{5}+\left|\text{tanh}(x)\right|,d_{\text{4}}(x)=\text{1}.\text{5}+\text{tanh}(x)$.

It can be observed from Figs 1–3, that the equilibrium point of system (1) is stable.

In Theorem 2, the parameter $\varrho_{\text{44}}$ is calculated as:


$$\varrho_{\text{44}}=\frac{\text{1}}{n}-\left|e_{\text{44}}\right|\sum_{k=\text{1}}^{\text{4}}\left(\left|e_{jk}\right|+\left|a_{jk}\right|+\left|b_{jk}\right|\right)=\frac{\text{1}}{\text{4}}-\text{4}(\text{6}e+\text{4}a+\text{4}b)=\frac{\text{1}}{\text{4}}-\text{24}e-\text{16}a-\text{16}b$$ (46)

For $e=\frac{\text{1}}{\text{15}}$, it follows that $\varrho_{\text{44}}<-\text{16}a-\text{16}b<\text{0}$. Hence, the conditions of Theorem 2 are not applicable to the case of the parameter values given in the example.

In Theorem 3, the parameter $e_{\text{4}}$ is calculated as:


$$e_{\text{4}}=\text{1}-\sum_{j=\text{1}}^{n}\left|e_{j\text{4}}\right|=\text{1}-\left|e_{\text{14}}\right|-\left|e_{\text{24}}\right|-\left|e_{\text{34}}\right|-\left|e_{\text{44}}\right|=\text{1}-\text{16}e$$ (47)

For $e=\frac{\text{1}}{\text{15}}$, it follows that $e_{\text{4}}=\text{1}-\text{16}e=-\frac{\text{1}}{\text{15}}<\text{0}$. Hence, the conditions of Theorem 3 are not applicable to the case of the parameter values given in the example.

In this example, we observe that the first stability condition set in Theorem 4 can be obtained as follows:


$$m_{\text{1}}=k_{\text{1}}-(a+b)\sum_{j=\text{1}}^{\text{4}}k_{j}>\text{0},\;m_{\text{2}}=k_{\text{2}}-(a+b)\sum_{j=\text{1}}^{\text{4}}k_{j}>\text{0},$$

$$m_{\text{3}}=k_{\text{3}}-(a+b)\sum_{j=\text{1}}^{\text{4}}k_{j}>\text{0},\;m_{\text{4}}=k_{\text{4}}-(a+b)\sum_{j=\text{1}}^{\text{4}}k_{j}>\text{0},$$ (48)

We note that, for this example, there are positive constants $k_{\text{1}},\;k_{\text{2}},\;k_{\text{3}}$ and $k_{\text{4}}\;$ for which $m_{\text{1}}>\text{0},\;m_{\text{2}}>\text{0},\;m_{\text{3}}>\text{0}$ and $m_{\text{4}}>\text{0}$ if I−(|A|+|B|) is determined to satisfy the nonsingular M-matrix condition (requiring that the real parts of the eigenvalues associated with this newly formed matrix I−|A|−|B| are positive, see reference [44], regarding some useful properties of M-matrices). For this example, I−|A|−|B| is obtained as


$$\begin{array}{c} I-|A|-|B|=\left[\;\;\;\begin{array}{cccc} \;1-(a+b) & -(a+b) & -(a+b) & -(a+b)\\ -(a+b) & 1-(a+b) & -(a+b) & -(a+b)\\ -(a+b) & -(a+b) & 1-(a+b) & -(a+b)\\ -(a+b) & -(a+b) & -(a+b) & 1-(a+b)\; \end{array}\;\;\;\;\;\right] \end{array}$$

If this formed matrix I−|A|−|B| possesses the nonsingular M-matrix property, then it is a fact that at least one column of I−|A|−|B| is strictly dominant [44]. Thus, this property of nonsingular M-matrices implies that 4(a+b)<1 and $k_{\text{1}}=\;k_{\text{2}}=\;k_{\text{3}}=k_{\text{4}}=\text{1}$. In this case, we can obtain the following equation for the conditions in Theorem 4:


$${\hat{e}}_{\text{4}}=\text{1}-\sum_{j=\text{1}}^{\text{4}}\left|e_{j\text{4}}\right|=\text{1}-\left|e_{\text{14}}\right|-\left|e_{\text{24}}\right|-\left|e_{\text{34}}\right|-\left|e_{\text{44}}\right|=\text{1}-\text{16}e$$ (49)

For $e=\frac{\text{1}}{\text{15}}$, it follows that ${\hat{e}}_{\text{4}}=\text{1}-\text{16}e=-\frac{\text{1}}{\text{15}}<\text{0}$. Thus, the conditions of Theorem 4 are not applicable to the case of the parameter values given in the example.

For the parameters of the given example, the conditions of Theorem 5 are largely identical to the conditions of Theorem 4. Therefore, the conditions $\sigma_{\text{1}}>\text{0},\;\sigma_{\text{2}}>\text{0},\;\sigma_{\text{3}}>\text{0}$ and $\sigma_{\text{4}}>\text{0}$ are satisfied if and only if 4(a+b)<1 and $k_{\text{1}}=\;k_{\text{2}}=\;k_{\text{3}}=k_{\text{4}}=\text{1}$. In this case, $\sigma \geq \frac{k_{M}\phi_{M}}{k_{m}\phi_{m}}=\text{1}$. Let σ=1. Then, we can observe that


$${\tilde{e}}_{\text{4}}=\text{1}-\sum_{j=\text{1}}^{\text{4}}\left|e_{j\text{4}}\right|=\text{1}-\left|e_{\text{14}}\right|-\left|e_{\text{24}}\right|-\left|e_{\text{34}}\right|-\left|e_{\text{44}}\right|=\text{1}-\text{16}e$$ (50)

For $e=\frac{\text{1}}{\text{1}\text{5}}$, it follows that ${\tilde{e}}_{\text{4}}=\text{1}-\text{1}\text{6}e=-\frac{\text{1}}{\text{1}\text{5}}<\text{0}$. Thus, the conditions of Theorem 5 are not applicable to the case of the parameter values given in the example.

In applying the conditions of Theorem 6 to the case of this example, we obtain


$$\pi_{\text{4}}=\text{2}-\left(\Omega_{\text{1}}+\Omega_{\text{2}}\right)-\frac{\text{1}}{\Omega_{\text{3}}}\psi_{\text{4}}^{\text{2}}-\left(\Omega_{\text{3}}+\Omega_{\text{4}}+\Omega_{\text{5}}\right)\sum_{j=\text{1}}^{n}\sum_{k=\text{1}}^{n}\left|e_{ji}e_{jk}\right|-\left(\frac{\text{1}}{\Omega_{\text{1}}}+\frac{\text{1}}{\Omega_{\text{4}}}\right)\sum_{j=\text{1}}^{n}\left|\sum_{k=\text{1}}^{n}a_{ki}a_{kj}\right|-\left(\frac{\text{1}}{\Omega_{\text{2}}}+\frac{\text{1}}{\Omega_{\text{5}}}\right)\sum_{j=\text{1}}^{n}\left|\sum_{k=\text{1}}^{n}b_{ki}b_{kj}\right|$$

$$\;=\text{2}-\left(\Omega_{\text{1}}+\Omega_{\text{2}}\right)-\frac{\text{1}}{\Omega_{\text{3}}}\psi_{\text{4}}^{\text{2}}-\left(\Omega_{\text{3}}+\Omega_{\text{4}}+\Omega_{\text{5}}\right)\text{96}e^{\text{2}}-\left(\frac{\text{1}}{\Omega_{\text{1}}}+\frac{\text{1}}{\Omega_{\text{4}}}\right)\text{16}a^{\text{2}}-\left(\frac{\text{1}}{\Omega_{\text{2}}}+\frac{\text{1}}{\Omega_{\text{5}}}\right)\text{16}b^{\text{2}}\;$$ (51)

For $\pi_{\text{4}}$, we can derive that $\pi_{\text{4}}<\text{2}-\frac{\text{1}}{\Omega_{\text{3}}}\psi_{\text{4}}^{\text{2}}-\Omega_{\text{3}}\text{96}e^{\text{2}}$. Note that $\frac{\text{1}}{\Omega_{\text{3}}}\psi_{\text{4}}^{\text{2}}+\Omega_{\text{3}}\text{96}e^{\text{2}}$ takes its minimum value when $\Omega_{\text{3}}=\frac{\psi_{\text{4}}}{\sqrt{\text{96}}e}$. In this case,


$$\pi_{\text{4}}<\text{2}-{\text{2}\psi }_{\text{4}}\sqrt{\text{96}}e=\text{2}-\text{48}e$$ (52)

For $e=\frac{\text{1}}{\text{15}}$, it follows that $\pi_{\text{4}}<\text{2}-\text{4}\text{8}e=-\frac{\text{1}\text{8}}{\text{1}\text{5}}<\text{0}$. Thus, the conditions of Theorem 6 are not applicable to the case of the parameters given in this example.

Based on the above comparisons, it can be concluded that this paper obtains some novel and alternative conditions that guarantee stability of Cohen-Grossberg systems with neutral model where the dynamical model representation of the system admits multiple delay parameters.

## 3. Conclusions

This paper examines the stability characteristics of neutral-type Cohen-Grossberg artificial neural networks. By making appropriate modifications to certain classes of Lyapunov functionals, novel sufficient algebraic conditions are formulated to guarantee the global asymptotic stability of Cohen-Grossberg neural systems with multiple constant delay parameters. It has been shown that the obtained stability criteria are of the forms of some algebraic equations that involve only some system parameters of the examined delayed Cohen-Grossberg neural network. Therefore, our proposed stability criteria can be checked by using various simple mathematical methods and software tools. By carrying out a detailed analysis of an instructive numerical example, the results obtained in this article are also shown to establish alternative stability criteria to some corresponding stability conditions given in the past literature.

## Supporting information

S1 Data This file archive contains the minimal data set required to replicate all study findings, including the raw numerical values used to generate the graphical representations of system responses under different network functions. All data files are provided in CSV format. (ZIP)
